# Integration of partial least squares and Monte Carlo gene expression analysis in coronary artery disease

**DOI:** 10.3892/etm.2014.1610

**Published:** 2014-03-07

**Authors:** HUAN ZHANG, TAO LI, GUANJI WU, FENG MA

**Affiliations:** Department of Cardiovascular Medicine, Xi’an Central Hospital, Xi’an, Shaanxi 710003, P.R. China

**Keywords:** coronary artery disease, gene expression, partial least squares, Monte Carlo

## Abstract

Coronary artery disease (CAD) is the most common type of cardiovascular disease and leading cause of mortality worldwide. Microarray technology for gene expression analysis has facilitated the identification of the molecular mechanism that underlies the pathogenesis of CAD. Previous studies have primarily used variance or regression analysis, without considering array specific factors. Thus, the aim of the present study was to investigate the mechanism of CAD using partial least squares (PLS)-based analysis, which was integrated with the Monte Carlo technique. Microarray analysis was performed with a data set of 110 CAD patients and 111 controls obtained from the Gene Expression Omnibus database. A total of 390 dysregulated genes were acquired. Significantly increased representations of dysregulated genes in Gene Ontology items, including transforming growth factor β-activated receptor activity and acyl-CoA oxidase activity, were identified. Network analysis revealed three hub genes with a degree of >10, including ESR1, ITGA4 and ARRB2. The results of the present study provide novel information on the gene expression signatures of CAD patients and offer further theoretical support for future therapeutic study.

## Introduction

Coronary artery disease (CAD) is the most common type of cardiovascular disease and leading cause of mortality worldwide (1). The disease is mainly caused by the build-up of plaque along the inner walls of heart arteries, which narrows the arteries and restricts blood flow. Typically, the majority of patients do not exhibit symptoms for decades in the progression of CAD. For the majority of individuals, the first onset of symptoms is acute myocardial infarction (heart attack). Numerous studies have been conducted with the aim of proposing promising strategies for the prevention and treatment of CAD. However, the morbidity and mortality rates of CAD remain high. Currently available high throughput experimental strategies aid the understanding of the pathogenic mechanism of CAD, constituting a significant advance for the development or improvement of novel strategies for the noninvasive diagnosis and treatment of CAD.

Previous gene expression studies (2–5) have proposed distinct gene expression patterns in CAD. Dysregulation of various biological processes, including the inflammatory process and cell cycle control, have been consistently detected in CAD patients (6,7). These studies used standard variance/regression analysis to identify differentially expressed genes. However, these analyses are unable to remove unaccounted array specific factors. For example, it is possible that specific genes are identified to be overexpressed or downregulated due to specific demographic profiles. A previous study (8) hypothesized that partial least squares (PLS)-based microarray analysis was robust in detecting disease specific genes. The PLS-based analysis method uses variable selection according to the analysis of the regression coefficients of PLS (9). Compared with variance/regression analysis, PLS-based analysis has higher sensitivity, reasonably high specificity and markedly smaller false discovery (FDR) and false non-discovery rates (8). In multiple regression analysis, the Monte Carlo cross-validation method is a powerful and widely used technique which was first reported by Picard and Cook (10). The use of Monte Carlo cross-validation in multiple regression analysis has been proposed in previous studies (11,12) and integration of the PLS and Monte Carlo technique is efficient in variable selection (13). Determining the gene expression signatures of CAD with the PLS-based method may further improve the understanding of the molecular mechanism and advance preventative or therapeutic procedures.

In the present study, using a microarray data set downloaded from the Gene Expression Omnibus (GEO) database, the pathological mechanism of CAD was investigated using PLS-based analysis. Gene Ontology (GO) items with significantly over-represented dysregulated genes were also acquired and protein-protein interaction (PPI) network analysis was performed to identify crucial genes among the dysregulated genes.

## Materials and methods

### Microarray data

The microarray data set, GSE12288, was downloaded from the GEO database (http://www.ncbi.nlm.nih.gov/geo/). This gene expression profile included 110 CAD patients and 112 healthy controls. The Duke CAD index (CADi) (14,15) was measured for each subject. The data set was based on the GPL96 platform: [HG-U133A] Affymetrix Human Genome U133A Array.

### Gene selection

Entire data sets for all the samples were downloaded. Robust multiarray analysis (16) was used to normalize the raw intensity values. Firstly, the effects of background noise and the processing artifacts were neutralized using model-based background correction. Secondly, expression values of all the probes were aligned to a common scale using quantile normalization. Finally, an expression value for each probe was generated via an iterative median polishing procedure. The resulting log2-transformed expression values were then used for subsequent analysis.

A multivariate linear model was used to analyze the association between gene expression levels and CADi. In the data set, the number of probes (n=22,283) was much greater than the sample number (n=221; one sample was deleted due to poor data quality). PLS, a dimension reduction procedure (17,18), was then used to estimate the effects for each gene. PLS latent variables derived from the expression profiles on CADi were calculated using the non-linear iterative PLS algorithm (19). Next, the variable importance on the projection (VIP) (20) was calculated to evaluate the importance of the genes on CADi. In addition, permutation tests were used to control the FDR. A permutation procedure (performed 1,000 times) was performed to obtain the empirical distribution of PLS-based VIP in each replicate. The FDR for each gene was evaluated based on the empirical distribution. Candidate genes were selected with a cut-off FDR value of <0.05.

The best number of latent variables was then determined using 4-fold cross-validation with root mean square error of cross-validation (RMSECV). Next, regression coefficient reliability was introduced using the Monte Carlo method (13). Firstly, 100 Monte Carlo sampling subsets, each of which included half of the total samples, were used to calculate the regression coefficient vector for each sub PLS model. Secondly, the regression coefficient reliability was calculated for each candidate gene. Regression coefficient reliability revealed not only a large coefficient, but also the stability of the genes for the disease. This was useful to alleviate deviation of the sampling and develop a robust disease prediction model with the most reliable target tag genes. An absolute value of regression coefficient reliability cut-off was selected according to the lowest RMSECV. Finally, genes that had absolute reliability values larger than the cut-off and FDR of VIP values <0.05 were selected as the target tag gene set.

### Enrichment analysis

Probes on the array were annotated according to the simple omnibus format in text files. To determine the biologically relevant signatures of the selected genes, enrichment analysis was performed. All the genes were annotated based on the GO database (21). The hypergeometric distribution test was then implemented to identify pathways that were significantly enriched with the selected genes.

### Network analysis

PPI is crucial for all biological processes (22). Selected genes that had a large number of interactions with other genes were considered to have more important roles in the pathogenesis. To visualize the interactions among the selected genes and identify the key molecules, a network was constructed using Cytoscape (V 2.8.3; http://www.cytoscape.org/) (23) and the National Center for Biotechnology Information database (http://ftp.ncbi.nlm.nih.gov/gene/GeneRIF/; accessed on the 25-2-2013). The degree of a gene was equal to the number of interactions the gene exhibited. Genes with a degree of >10 were considered as critical hub molecules.

## Results

### Gene selection

Gene expression profiles of 110 CAD patients and 111 healthy controls (one sample was deleted due to low quality) were used for subsequent analysis. PLS analysis revealed that 1,246 genes were considered as candidate genes with FDR of VIP values of <0.05. To avoid model over-fitting, the best number of latent variables was then determined by 4-fold cross-validation with RMSECV. The results indicated that RMSECV values exhibited a descending trend with an increase in latent variable number, however, the trend decreased in strength with latent variable numbers of >8. Therefore, the top eight latent variables were selected for further analysis. Regression coefficient reliability of each candidate gene was calculated and the cut-off value (1.434) was determined according to the lowest RMSECV value. In total, 390 genes were selected.

### Enrichment analysis

Table I represents the top five GO items enriched with the selected genes. Of all the genes in the array, 12,291 genes were annotated based on the GO database, including 358 selected genes. Items with significantly increased representations of the selected genes included transforming growth factor β-activated receptor (TGFBR) activity, acyl-CoA oxidase activity, transcription regulatory region sequence-specific DNA binding, erythrocyte differentiation and negative regulation of the mitogen-activated protein kinase cascade.

### Network analysis

**Figure 1:**
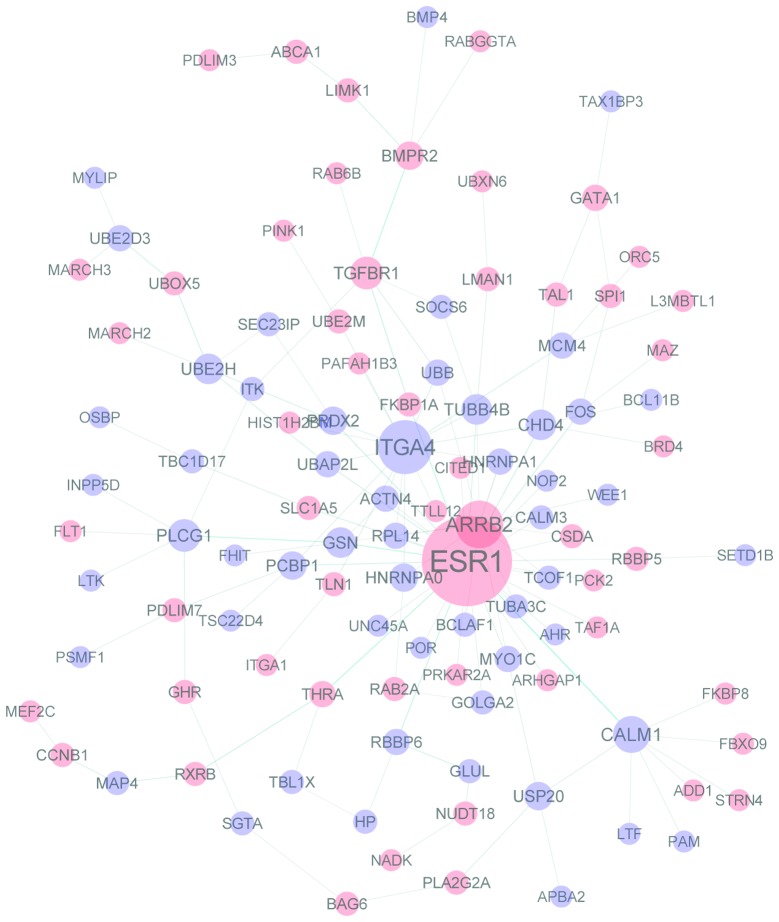
Interaction network constructed with the identified differentially expressed genes. Only genes with more than two direct or indirect associations are shown. Genes with a higher degree (more associations or interactions) are shown as a larger size. Genes shown in pink are overexpressed, while genes in blue are downregulated.

Fig. 1 illustrates the interaction network of the proteins encoded by the selected genes. Hub molecules that had a degree of >10 included ESR1, ITGA4 and ARRB2, with degrees of 33, 16 and 13, respectively.

## Discussion

CAD is a highly complex disease. Gene expression profiling is important for investigating the underlying pathophysiological cascades in CAD. For data analysis, a suitable model is required to manage the large number of genes and the relatively small sample size. Previous gene expression studies on CAD primarily used variance/regression analysis, without considering the hidden biological effects. In the present study, an integration of the PLS and Monte Carlo technique (13) was used to identify differentially expressed genes in CAD. Biological process and interaction network analysis were also used to explore the underlying mechanism.

GO item enrichment analysis revealed that TGFBR activity (GO:0005024) was the most significant GO item with over-represented dysregulated genes (Table I). All the dysregulated genes in this item were upregulated in CAD patients, including TGFBR1. A previous study revealed that the inhibition of TGFBR1 results in significant amelioration of deleterious cardiac remodeling following myocardial infarction (24). The results of the present study indicated that TGFBR1 and other TGFBRs may function as potential targets for further treatment investigation studies. Significantly increased numbers of dysregulated genes were also identified in the acyl-CoA oxidase activity item. A previous study (25) hypothesized that supplementation with polyphenolic-rich extract of *Angelica acutiloba* root for high-fat diet-induced obese rats significantly decreased the CAD risk index by enhancing the expression of acyl-CoA oxidase. The results of the present study confirmed the involvement of acyl-CoA oxidase in the pathogenesis of CAD.

Interaction network analysis revealed that ESR1 was the hub gene with the highest degree (Fig. 1). The protein encoded by this gene is the estrogen receptor. Genetic polymorphisms of ESR1 have been shown to be associated with CAD in various populations (26–28) and the results of the present study confirmed the involvement of ESR1 in CAD. ITGA4 was also identified as a hub gene with the second highest degree (Fig. 1). The protein encoded by this gene belongs to the integrin α chain family of proteins. No previous studies have proposed an association between CAD and ITGA4, however, gene targeting experiments in mice have demonstrated an essential role of ITGA4 in normal epicardial development (29). Therefore, the association between ITGA4 and CAD requires further investigation. ARRB2 was also identified as a hub gene with a degree of 13. The protein encoded by this gene belongs to the arrestin/β-arrestin protein family. Similarly, no previous studies have proposed an association between CAD and ARRB2, thus, further study is required to investigate the involvement of this gene in the pathogenesis of CAD.

In conclusion, using a gene expression microarray data set downloaded from the GEO database, PLS-based analysis integrated with the Monte Carlo technique was performed to identify genes which may contribute to the pathology of CAD. Further analysis was also conducted to identify biological processes and hub genes associated with the disease. Therefore, the results of the present study facilitate the disclosure of the molecular mechanism underlying CAD.

## Figures and Tables

**Table I:** Top five GO items enriched with the selected genes.

GO identification	Description	P-value
0005024	TGFBR activity	8.08E-06
0003997	Acyl-CoA oxidase activity	1.01E-05
0000976	Transcription regulatory region sequence-specific DNA binding	1.63E-05
0030218	Erythrocyte differentiation	3.01E-05
0043409	Negative regulation of the MAPK cascade	4.11E-04

GO, Gene Ontology; MAPK, mitogen-activated protein kinase; TGFBR, transforming growth factor β-activated receptor.

## References

[1] Thomas AC, Knapman PA, Krikler DM, Davies MJ (1988). Community study of the causes of ‘natural’ sudden death. BMJ.

[2] Hiltunen MO, Tuomisto TT, Niemi M (2002). Changes in gene expression in atherosclerotic plaques analyzed using DNA array. Atherosclerosis.

[3] Nanni L, Romualdi C, Maseri A, Lanfranchi G (2006). Differential gene expression profiling in genetic and multifactorial cardiovascular diseases. J Mol Cell Cardiol.

[4] Randi AM, Biguzzi E, Falciani F (2003). Identification of differentially expressed genes in coronary atherosclerotic plaques from patients with stable or unstable angina by cDNA array analysis. J Thromb Haemost.

[5] Seo D, Wang T, Dressman H (2004). Gene expression phenotypes of atherosclerosis. Arterioscler Thromb Vasc Biol.

[6] Cagnin S, Biscuola M, Patuzzo C (2009). Reconstruction and functional analysis of altered molecular pathways in human atherosclerotic arteries. BMC Genomics.

[7] Sluimer JC, Kisters N, Cleutjens KB (2007). Dead or alive: gene expression profiles of advanced atherosclerotic plaques from autopsy and surgery. Physiol Genomics.

[8] Chakraborty S, Datta S, Datta S (2012). Surrogate variable analysis using partial least squares (SVA-PLS) in gene expression studies. Bioinformatics.

[9] Centner V, Massart DL, de Noord OE, de Jong S, Vandeginste BM, Sterna C (1996). Elimination of uninformative variables for multivariate calibration. Anal Chem.

[10] Picard RR, Cook RD (1984). Cross-validation of regression models. J Am Stat Assoc.

[11] Xu QS, Liang YZ, Du YP (2004). Monte Carlo cross-validation for selecting a model and estimating the prediction error in multivariate calibration. J Chemom.

[12] Gourvénec S, Fernández Pierna JA, Massart DL, Rutledge DN (2003). An evaluation of the PoLiSh smoothed regression and the Monte Carlo cross-validation for the determination of the complexity of a PLS model. Chemometr Intell Lab Syst.

[13] Cai WS, Li YK, Shao XG (2008). A variable selection method based on uninformative variable elimination for multivariate calibration of near-infrared spectra. Chemometr Intell Lab.

[14] Felker GM, Shaw LK, O’Connor CM (2002). A standardized definition of ischemic cardiomyopathy for use in clinical research. J Am Coll Cardiol.

[15] Mark DB, Nelson CL, Califf RM (1994). Continuing evolution of therapy for coronary artery disease. Initial results from the era of coronary angioplasty. Circulation.

[16] Irizarry RA, Hobbs B, Collin F (2003). Exploration, normalization, and summaries of high density oligonucleotide array probe level data. Biostatistics.

[17] Helland IS (1988). On the structure of partial least squares regression. Commun Stat-Simulation Comput.

[18] Helland IS (1990). Partial least squares regression and statistical model. Scand J Stat.

[19] Martins JPA, Teófilo RF, Ferreira MMC (2010). Computational performance and cross-validation error precision of five PLS algorithms using designed and real data sets. J Chemom.

[20] Gosselin R, Rodrigue D, Duchesne C (2010). A bootstrap-VIP approach for selecting wavelength intervals in spectral imaging applications. Chemometr Intell Lab Syst.

[21] Ashburner M, Ball CA, Blake JA, The Gene Ontology Consortium (2000). Gene ontology: tool for the unification of biology. Nat Genet.

[22] Stelzl U, Worm U, Lalowski M (2005). A human protein-protein interaction network: a resource for annotating the proteome. Cell.

[23] Shannon P, Markiel A, Ozier O (2003). Cytoscape: a software environment for integrated models of biomolecular interaction networks. Genome Res.

[24] Ellmers LJ, Scott NJ, Medicherla S (2008). Transforming growth factor-beta blockade down-regulates the renin-angiotensin system and modifies cardiac remodeling after myocardial infarction. Endocrinology.

[25] Liu IM, Tzeng TF, Liou SS, Chang CJ (2012). Regulation of obesity and lipid disorders by extracts from *Angelica acutiloba* root in high-fat diet-induced obese rats. Phytother Res.

[26] Almeida S, Hutz MH (2006). Estrogen receptor 1 gene polymorphisms and coronary artery disease in the Brazilian population. Braz J Med Biol Res.

[27] Lawlor DA, Timpson N, Ebrahim S, Day IN, Smith GD (2006). The association of oestrogen receptor alpha-haplotypes with cardiovascular risk factors in the British Women’s Heart and Health Study. Eur Heart J.

[28] Wei CD, Zheng HY, Wu W (2013). Meta-analysis of the association of the rs2234693 and rs9340799 polymorphisms of estrogen receptor alpha gene with coronary heart disease risk in Chinese Han population. Int J Med Sci.

[29] Dettman RW, Pae SH, Morabito C, Bristow J (2003). Inhibition of alpha4-integrin stimulates epicardial-mesenchymal transformation and alters migration and cell fate of epicardially derived mesenchyme. Dev Biol.

